# Trajectories of adolescent conduct problems in relation to cortical thickness development: a longitudinal MRI study

**DOI:** 10.1038/tp.2016.111

**Published:** 2016-06-21

**Authors:** S Oostermeijer, S Whittle, C Suo, N B Allen, J G Simmons, N Vijayakumar, P M van de Ven, L M C Jansen, M Yücel, A Popma

**Affiliations:** 1 VU University Medical Centre Amsterdam, Child and Adolescent Psychiatry, Amsterdam, The Netherlands; 2 Department of Psychiatry, Melbourne Neuropsychiatry Centre, The University of Melbourne and Melbourne Health, Melbourne, VIC, Australia; 3 Brain and Mental Health Laboratory, School of Psychological Sciences and Monash Biomedical Imaging Facility, Monash University, Melbourne, VIC, Australia; 4 Melbourne School of Psychological Sciences, The University of Melbourne, Melbourne, VIC, Australia; 5 Orygen, The National Centre of Excellence in Youth Mental Health, Melbourne, VIC, Australia; 6 Department of Psychology, University of Oregon, Eugene, OR, USA; 7 Department of Epidemiology and Biostatistics, VU University Medical Centre Amsterdam, Amsterdam, The Netherlands; 8 Institute of Criminal Law and Criminology, Leiden University, Leiden, The Netherlands

## Abstract

Multiple cross-sectional imaging studies have identified structural abnormalities in prefrontal, temporal and limbic regions related to conduct problems (CPs). However, the relationship between development of such neurobiological deficits and developmental pathways of CPs has remained unclear. The current study investigated distinct trajectories of CP and related trajectories of cortical thickness within a community-based sample of adolescents (*n*=239), age range 12–19, to address this gap. Three trajectory classes were revealed using latent class growth analyses (LCGAs), comprising a ‘desisting' CP group, an ‘intermediate' CP group and a ‘stable low' CP group. Structural magnetic resonance imaging (MRI) scans were collected with a subgroup of 171 adolescents at three waves throughout adolescence (ages 12, 16 and 19). Generalized estimating equation (GEE) analysis—comparing longitudinal changes in cortical thickness and subcortical volume between CP groups for several regions of interest (ROIs)—showed that these CP groups had differential trajectories of cortical thickness in the dorsolateral prefrontal cortex (dl-PFC), and the anterior cingulate cortex (ACC), and volume of the hippocampus. Adolescents in the desisting CP group showed an attenuation of the typical pattern of cortical thinning as present in the intermediate and stable low CP groups, in addition to an exaggeration of the typical pattern of hippocampal volume increase. These findings suggest that a deviant cortical thickness trajectory was related to a desisting CP pathway across adolescence. Such deviant neurodevelopmental growth trajectories may act as an underlying mechanism for developmental CP pathways, and possibly distinguish desisting antisocial adolescents.

## Introduction

Severe antisocial behavior in adolescents predicts a variety of negative life outcomes and brings considerable costs for society.^1, 2, 3^ Unraveling the underlying mechanisms of antisocial behaviors might contribute to a better understanding of factors that influence their development, and hence development of more effective and tailored intervention and prevention strategies. Investigation of brain development might be fruitful in this regard. A wide range of studies identifying distinct trajectories of antisocial behavior with different techniques, samples and follow-up lengths have consistently shown an adolescent-limited (that is, desistant) and chronic group (that is, persistent).^3, 4^ However, most studies differ in additional trajectories detected, such as intermediate groups and late-onset chronic groups.^4^ For example, an epidemiological study on the validity of developmental conduct problems (CP; which typically define antisocial behavior in children and adolescents) showed 4 classes of CP trajectories in males; a life-course persistent-, an adolescent onset-, childhood-limited- and a low CP class.^3^

Neurobiological correlates of antisocial behaviors have become an apparent field of research. A meta-analysis of structural as well as functional imaging studies in antisocial adults and children reported volumetric reductions in a range of brain regions, particularly in the orbitofrontal cortex (OFC), the dorsolateral prefrontal cortex (dl-PFC) and the anterior cingulate cortex (ACC).^5^ Studies have also reported reduced insula volume in adolescents with CPs compared with healthy controls.^6, 7, 8^ Researchers have mainly been focused on the *early-onset* type (age <12) of antisocial behavior defined as early-onset conduct disorder (CD).^6, 7^^9^ However, Fairchild *et al.*^8^ have shown that CD is associated with gray matter abnormalities regardless of the age of onset. In addition, CD symptomatology has been inversely correlated with gray matter volumes in limbic structures (including the temporal pole, amygdala, middle temporal gyrus, parahippocampal gyrus, medial temporal lobe, hippocampus and the inferior temporal gyrus) and prefrontal areas (superior medial gyrus and middle frontal gyrus).^6^ A study comparing pre-adolescent CD boys to typical developing boys, however, reported increased volumes in the medial OFC and ACC, and no regions of decreased gray matter concentration or volume were detected.^9^ A recent study with CD adolescents showed that high levels of CD symptoms were associated with reduced gray matter volume in the temporal region around the superior temporal sulcus.^10^ As such, it remains unclear whether CPs are associated with increased or decreased gray matter volumes.

There has been growing interest in psychopathic traits in children and their association with development and persistence of antisocial behavior. These psychopathic traits have been associated with more CD symptoms and earlier onset of CD, as well as more delinquency and higher recidivism in juveniles.^11, 12^ Research on psychopathy indicates that frontal structures and paralimbic areas have an important role, including the ACC, insula and parahippocampal gyrus.^13, 14^ A more recent study by Yang *et al.*^15^ showed cross-sectional and longitudinal correlations between psychopathic scores over time and frontal and temporal cortical thicknesses at the age of 14 in healthy at-risk adolescents. Longitudinal magnetic resonance imaging (MRI) studies toward brain development and the development of antisocial behavior (including CPs and psychopathic traits) are warranted, but currently lacking. De Brito *et al.*^9^ suggested that gray matter concentration differences in their regions of interest (ROIs; medial OFC and dorsal ACC) reflected a maturational lag in CD boys. This could possibly explain conflicting results in cross-sectional imaging studies, with varying age ranges from pre- to late-adolescent samples. Cortical abnormalities have also been shown to have a role in more subtle forms of CPs present in the community, as opposed to adolescents diagnosed with CD specifically.^16, 17, 18^

This study aimed to investigate whether distinct CP pathways showed different growth trajectories of cortical thickness from early to late adolescence. Neurodevelopmental trajectories can go awry in several ways, and deviations in these trajectories may confer risk or vulnerability to mental illness. Such deviant trajectories may have the same form as a typical trajectory but delayed, they may change at a disrupted velocity or loose the form or shape of a typical trajectory.^19^ For example, clinical outcome in attention-deficit/hyperactivity disorder (ADHD) with delayed, but eventually normalizing, cortical thickness has been related to decreases in ADHD symptoms.^19^ As such, it could be of clinical relevance to unravel deviant brain developmental trajectories in relation to antisocial behavior across adolescence. Longitudinal data from a community-based sample were used to identify homogeneous classes of different CP pathways. Psychopathic traits were assessed to validate antisocial pathways. To ensure adequate power, the CP classes were compared with respect to development of a single parameter, that is, cortical thickness, in *a priori* defined ROIs. We hypothesized that four developmental classes of CP would be found similar to earlier research, with a persistent CP group showing deviant cortical growth trajectories. In addition, *post hoc* analyses investigated surface area and gyrification of aforementioned ROIs and subcortical volume of the amygdala and the hippocampus (see Supplementary Data).

## Materials and methods

### Participants

**Figure 1:**
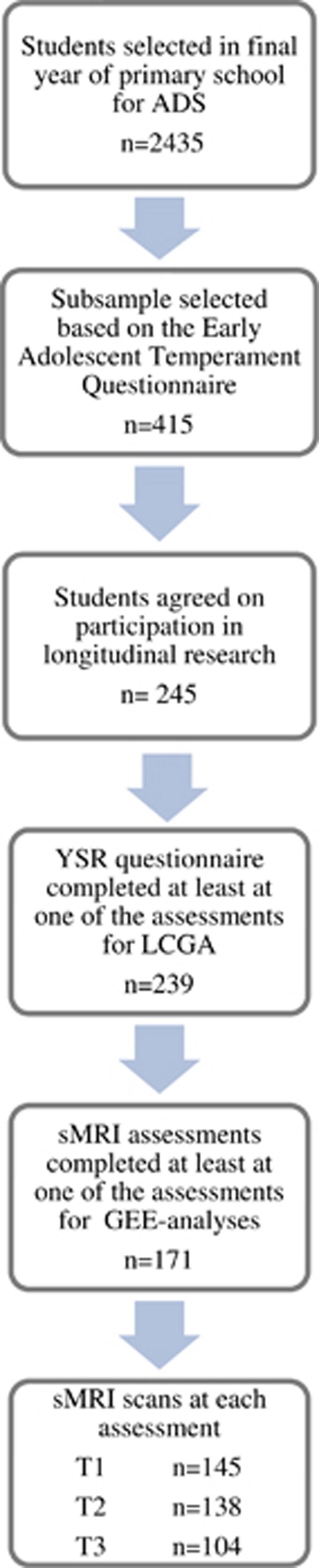
Sample selection. *Note:* Adolescent Development Study (ADS) from Melbourne, Australia, see Yap *et al.*^21^ GEE, generalized estimated equation; sMRI, structural magnetic resonance imaging; YSR, Youth Self Report.

A subsample was derived from the Orygen Adolescent Development Study, conducted in Melbourne, Australia (see Figure 1). On the basis of the Early Adolescent Temperament Questionnaire-Revised^20^, students in their final year of primary school were selected, previously described by Yap *et al.*^21^ Children at extreme ends of the temperamental distribution were oversampled. Children who had no chronic illness, language or learning disabilities and did not use medication known to affect nervous system functioning were asked to take part in longitudinal research and brain MRI assessments. There were three assessment waves at the approximate ages of 12 (T1), 16 (T2) and 19 (T3). Timing of T2 differed for questionnaire and MRI assessments. On average, MRI assessment was 19.7 months (s.d. 5.7) later as the questionnaire-based assessment. This was not deemed problematic for further analysis, as these measurements were used for separate trajectory analyses. Intelligence was assessed at T1 by the short form of the Wechler Intelligence Scale for Children. Socioeconomic status (SES) was estimated using the Australian National University Four^22^ (ANU4), ranging from 0 to 100. Participants were screened with the Kiddie Schedule for Affective Disorder and Schizophrenia for School-Aged Children: Present and Lifetime Version^23^ (K-SADS) at all assessment waves for CD and oppositional defiant disorder. Informed consent was obtained from the child and at least one parent/guardian at each assessment, consistent with the guidelines of the Human Research Ethics Committee at the University of Melbourne, Australia.

### Antisocial behavior

CPs were measured using the Youth Self-Report questionnaire (YSR version 2001). Good psychometric properties have been reported.^24^ Each item is answered with a Likert scale consisting of ‘not true (1)', ‘sometimes/somewhat true (2)' or ‘often/totally true (3)'. The scale ‘CPs' was used for identifying homogenous classes of CPs. Converted *T*-scores between 65 and 69 indicate subclinical problems, and *T*-scores above 69 indicate clinically relevant problems.

The Antisocial Personality Screening Device^25^ was used to assess psychopathic traits. It consists of 20 self-report items each answered with a Likert scale consisting of ‘not at all true (0)', ‘sometimes true (1)' or ‘definitely true (2)'. Validity and reliability, particularly for the total Antisocial Personality Screening Device score, has been demonstrated.^26^

### Internalizing problems

Internalizing problems were measured by two self-report questionnaires separately assessing anxiety and depression. First, the Beck Anxiety Inventory^27^ was used to measure state anxiety at all assessment waves. It consists of 21 self-report items, each answered with a Likert scale consisting of ‘rarely or never (0)', ‘occasionally (1)', ‘often (2)' or ‘almost always (3)'. Good validity and reliability have been shown including in nonclinical samples.^28^ Further, the Center for Epidemiologic Studies Depression Scale^29^ was used to measure depressive symptoms. It consists of 20 self-report items each answered with a Likert scale consisting of ‘rarely (1)', ‘some of the time (2)', ‘occasionally (3)' or ‘most of the time (4)'. The Center for Epidemiologic Studies Depression Scale has been shown to be valid and reliable in adolescents.^30^

### Image acquisition and processing

At the first assessment, MRI scans were performed on a 3-T GE scanner with the following parameters, repetition time=36 ms, echo time=9 ms, flip angle=35°, field of view=20 cm^2^, to obtain 124 T1-weighted continuous slices (voxel dimensions=0.4883 × 0.4883 × 1.5 mm). At the second and third assessments, MRI scans were performed on a 3-T Siemens scanner with the following parameters, repetition time=1900 ms, echo time=2.24 ms, flip angle=9°, field of view= 23 cm^2^, to obtain 176 T1-weighted continuous 0.9-mm-thick slices (voxel dimensions=0.9 mm^3^).

The stability of image acquisition may be compromised because of instrument-related differences between sites and instruments or software updates. As described in earlier studies with the current sample, steps were taken to address two main sources of error (that is, geometric distortion and voxel dimension drifts^31, 32^). First, images were corrected for tissue signal inhomogeneity. This was achieved using a nonparametric nonuniformity intensity normalization method optimized for 3-T images using Freesurfer's N3 correction. Second, voxel dimension drift was corrected using linear registration procedures used by the longitudinal processing stream in FreeSurfer 5.3 (http://surfer.nmr.mgh.harvard.edu/fswiki/LongitudinalProcessing), which involves the creation of an unbiased within-subject template space and average image using robust, inverse consistent registration. With regards to interscanner bias, former studies have performed an inter-scanner study for which four individuals were scanned on both scanner platforms within a 3-week period to address this issue. These studies have shown minimal to no effect on structural MRI data with regards to an interscanner bias.^31, 32, 33^ It has repeatedly been shown that the volume/thickness differences found between scanners were not notably different to within-scanner estimates that have been described previously.^34^ More importantly, interscanner bias will not affect the comparison between groups.

### ROIs

The following cortical regions were selected for investigation based on previous research on antisocial behaviors: the OFC, the dl-PFC, the ACC, the insula and the parahippocampal area. First, the cortical thickness of each region was estimated at each assessment wave using the output from FreeSurfer longitudinal stream. The individual's cortical reconstruction was visually inspected for all ROIs by a trained researcher to ensure that optimal gray/white and gray/cerebrospinal fluid classification had occurred based on differences in tissue intensity signals. Manual edits were made where necessary to ensure/improve quality of the output. For each hemisphere, 34 cortical parcellation units were automatically identified and labeled, as part of FreeSurfer's automatic cortical parcellation routine, according to the Desikan atlas of gyral-based definitions.^35^ Second, for each hemisphere, the mean thickness of the OFC was created by combining the medial and lateral OFC parcellation units, the ACC by combining the rostral and caudal ACC and the dl-PFC by combining the superior frontal, rostral middle frontal and caudate middle frontal cortex. In addition, volumes of the amygdala and the hippocampus were investigated. Volumes were estimated based on FreeSurfer's automated segmentation.^36^

### Statistical analysis

#### Latent class growth analysis

A latent class growth analysis (LCGA) was preformed on the YSR CP scale in M-plus version 6.11. In total, 239 adolescents were included who had completed the YSR CP scale at least at one out of three assessment waves. Each individual was classified into a distinct group based on the individual CP response pattern over time. To investigate whether missing data influenced the results, the LCGA was rerun excluding adolescents with missing data. Selection of the number of classes was guided by the Bayesian Information Criterion, the bootstrap likelihood ratio test, the Lo, Mendell and Rubin likelihood ratio test, and the entropy, together with the size of classes and interpretability of the classification.^37^ Longitudinal patterns of the mean converted T-scores on the CP scale within each of the classes were used to investigate clinical scores. Furthermore, longitudinal patterns of the mean psychopathic traits were used to validate classification based on CP scores. In addition, for each of the LCGA class, participants were checked for CD and Oppositional Defiant Disorder results (Supplementary Figure 1).

#### Regions of interest

Developmental trajectories of cortical thickness/subcortical volume of ROIs were compared between CP-based LCGA classes using generalized estimating equation (GEE) analysis performed in SPSS version 22 (Melbourne, VIC, Australia). Correlations between repeated thickness/volume measurements of the same individual were taken into account by using an exchangeable (working) correlation structure. The cortical thickness or subcortical volume of each ROI was introduced as the dependent variable. The regression model contained the age at measurement and LCGA class as independent variables, together with their interaction. The main interest was in testing whether changes in cortical thickness/subcortical volume over time differed between the classes by testing the interaction for significance. Separate GEE analyses were performed for different ROIs, left and right separately. Significance level was set at 5% for all analyses. All analyses were corrected for SES, gender and the interaction between gender and age to account for gender- and SES-related differences in the mean cortical thickness and gender-related differences in changes in thickness over time. Finally, to address specificity of results to CPs, longitudinal internalizing (that is, depression and anxiety) symptoms were included as covariates in a second set of analyses.

## Results

### LCGA

Two-, three- and four-class LCGA models were estimated. The four-class solution ended up with a class consisting of three individuals, and therefore the number of classes was not further increased. On the basis of the different fit indices, the four-class solution seemed the most suitable as it had significant Lo, Mendell and Rubin likelihood ratio test and bootstrap likelihood ratio test, lowest Bayesian Information Criterion and highest entropy (Table 1A). However, visual inspection of the growth trajectories indicated that the four-class solution was very similar to the three-class solution. On the basis of this observation, the small number of individuals in the fourth class (*n*=3) and careful theoretical consideration, the three-class solution was selected for further analysis. The LCGA was rerun with individuals, who completed all assessments, to check whether missing data influenced results. This sensitivity analysis revealed the three-class solution as the best fitting model (Table 1B), and trajectories were similar to those found using all data.

**Figure 2:**
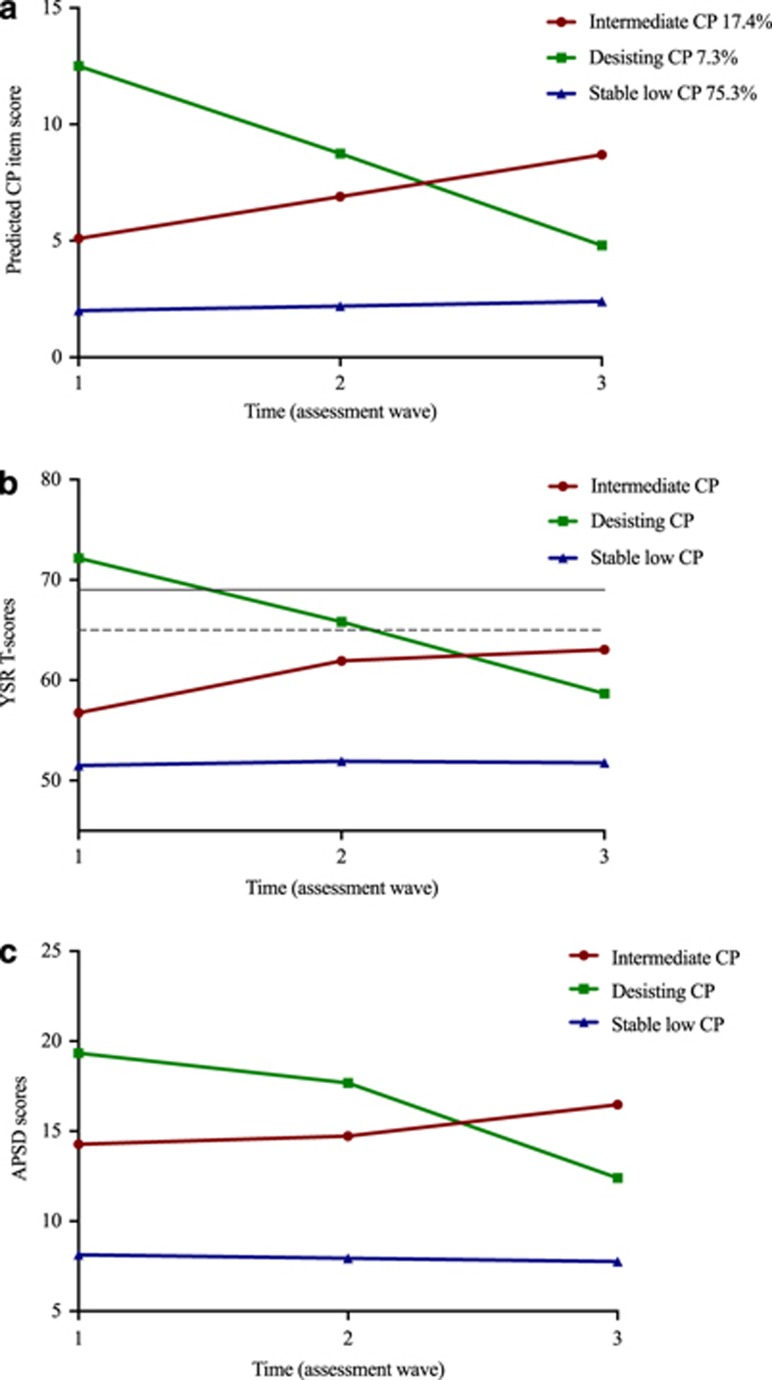
Antisocial behavioral scores of the three conduct problem (CP) trajectory classes. (**a**) Mean CP-scores on the YSR for the LCGA classes per assessment wave. (**b**) T-Scores of the YSR for the three classes at each assessment wave. *Note*: Dotted line represents the sub-clinical cut-off and the solid line represents the clinical cut-off of the YSR conduct problem T-scores. (**c**) Scores of the Antisocial Personality Screening Device (APSD) for the three classes at each wave. LCGA, latent class growth analyses; YSR, Youth Self-Report.

Trajectories of the mean CP scores in each of the LCGA classes are shown in Figure 2a. The CP trajectories found are referred to as follows: the intermediate CP group (class 1), the desisting CP group (class 2) and the stable low CP group (class 3). Results showed absence of a severe persistent CP group in the current sample. The mean T-scores from the YSR CP of the classes are plotted in Figure 2c, showing a similar pattern. When re-running LCGA analyses including CU scores and CP scores, this resulted in similar classes and there was no indication that CU was of importance in subtyping CP groups. In addition, all Antisocial Personality Screening Device scales correlated highly with the CP scores (varying between 0.394–0.775, with *P*<0.001).

### GEE analyses

A total of 171 adolescents took part in both MRI- and questionnaire-based assessment on at least one of the three assessment waves. The LCGA allocated each adolescent to one out of the three classes representing a distinct pattern of their CP trajectory, see Table 2 for descriptives per class.

**Figure 3:**
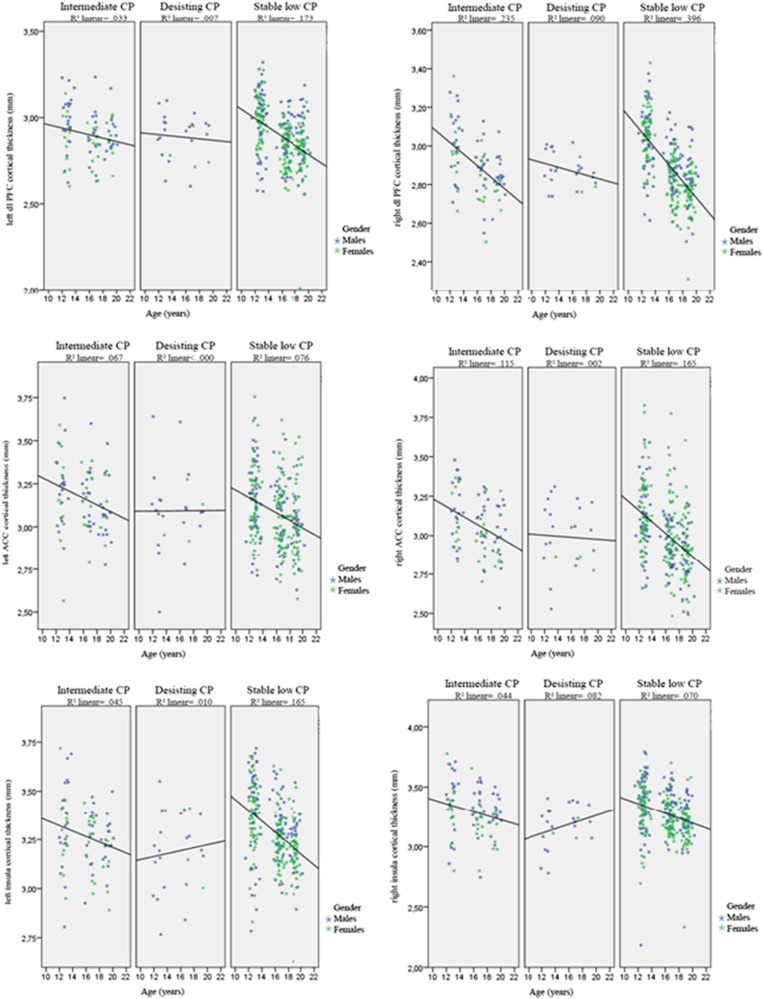
Uncorrected cortical thickness of the left and right dorsolateral prefrontal cortex (dl-PFC), anterior cingulate cortex (ACC) and insula with linear fitted lines. 1= the intermediate conduct problem group, 2= the desisting conduct problem group, 3= the stable low conduct problem group. CP, conduct problem.

Changes in cortical thickness in the OFC and parahippocampal area over time did not differ between the CP groups. A significant interaction between age and CP group was found for the right dl-PFC (Wald's, *X*^2^=27.73, degree of freedom (df)=2, *P*<0.001), and the left dl-PFC showed a trend (Wald's *X*^2^=5.69, df=2, *P*=0.058). Plotted results for the dl-PFC for the three CP groups (Figure 3) indicated a less steep decline over time for the desisting CP group compared with the other CP groups. In addition, the right dl-PFC showed a significant interaction effect of gender and age (Wald's *X*^2^=5.87, df=1, *P*<0.05). A significant interaction between age and CP group was found for the left (Wald's, *X*^2^=9.30, df=2, *P*<0.010) and right (Wald's, *X*^2^=12.44, df=2, *P*<0.005) ACC and the left (Wald's, *X*^2^=6.68, df=2, *P*<0.05) and right (Wald's, *X*^2^=7.35, df=2, *P*=0.025) insula. Plotted results of the ACC for the three CP groups (Figure 3) indicated a less steep decline over time for the desisting CP group compared with the other CP groups. For the insula (Figure 3), plotted results indicated a slight increase for the desisting CP group, whereas the other CP groups show a decline. After applying a Bonferroni correction for multiple comparisons (*P*<0.004), the interaction effect of age and class remained significant for the right dl-PFC and right ACC.

**Figure 4:**
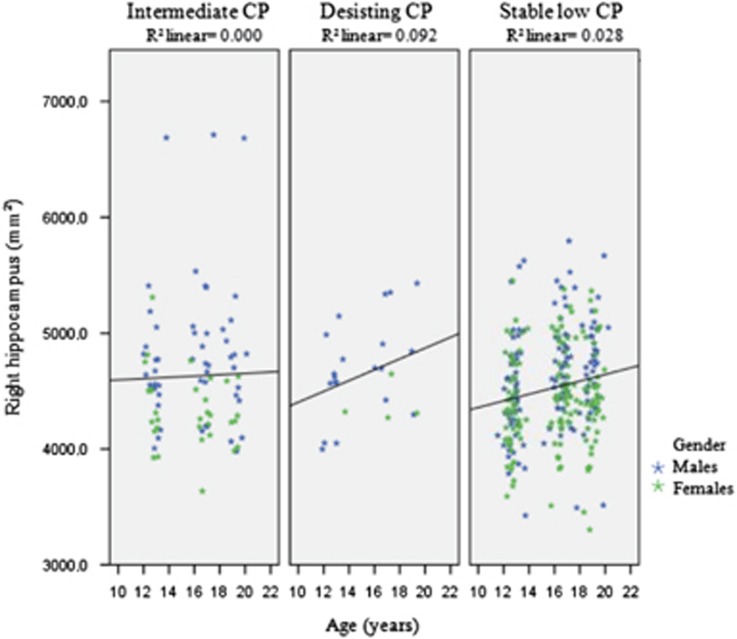
Uncorrected subcortical volume of the right hippocampus with linear fitted lines. 1= the intermediate conduct problem group, 2= the desisting conduct problem group, 3= the stable low conduct problem group. CP, conduct problem.

Results showed that changes in subcortical volume of the right and left amygdala over time did not differ between CP groups. In addition, no interaction effects were observed. For the hippocampus, the results showed changes in volume in the left hemisphere over time that did not differ between the CP groups. There was a significant interaction effect between gender and age (Wald's, *X*^2^=7.15, df=1, *P*<0.01). For the right hippocampus, results showed changes in subcortical volume over time that significantly differed between the CP groups (Wald's, *X*^2^=11.01, df=2, *P*<0.005). In addition, a significant interaction effect of gender and age was found (Wald's, *X*^2^=6.46, df=1, *P*<0.01). Plotted results for the right hippocampus for the three CP groups (Figure 4) indicated a greater increase for the desisting compared with the other CP groups. After applying a Bonferroni correction for multiple comparisons (*P*<0.004), the interaction effect of age and class remained significant for the right hippocampus.

### Internalizing problems

Correcting for internalizing problems (that is, anxiety and depressive symptoms) did not substantially influence results. Similarly to the primary analyses, a significant interaction between age and CP group was found for the right dl-PFC (Wald's, *X*^2^=16.85, df=2, *P*<0.000), and a trend for the left PFC was found (Wald's, *X*^2^=4.73, df=2, *P*=0.094). Similarly to the primary analyses, a significant interaction between age and CP group was found for the right ACC (Wald's, *X*^2^=8.15, df=2, *P*<0.05) and right insula (Wald's, *X*^2^=6.21, df=2, *P*<0.05). However, previous significant results for the left ACC and left insula were reduced to trend-level significance (left ACC: Wald's, *X*^2^=5.90, df=2, *P*=0.052; left insula: Wald's, *X*^2^=5.41, df=2, *P*=0.067). Finally, the significant interaction between age and CP group remained for the right hippocampus (Wald's, *X*^2^=8.33, df=2, *P*<0.05) when correcting for internalizing problems.

## Discussion

The present study revealed three CP trajectories during adolescence in a community-based sample: a desisting CP group, an intermediate CP group and a stable low CP group. These antisocial trajectories were validated with psychopathic scores throughout adolescence. The CP groups showed differential trajectories of cortical thickness in the dl-PFC, ACC and insula. Our hypothesis was partly confirmed, finding CP trajectories in line with earlier research; however, no persistent CP group was identified. A deviant cortical growth trajectory was observed in the desisting CP group.

The desisting CP group demonstrated severe CPs in early adolescence, with converted clinical scores in the clinical range, desisting through late adolescence, similar to the childhood-limited class identified by Odgers *et al.*^3^ The largest group showed a stable low CP pattern throughout adolescence, which was confirmed by their normative scores on the converted clinical scores of the CP scale. The trajectory of CP in the intermediate group, an elevated pattern of CPs throughout adolescence, is less straightforward to interpret. This intermediate CP group could arguably be labeled as ‘adolescent onset' or a persistent group. However, the mean CPs remained below the (sub) clinical threshold of converted scores throughout adolescence. In addition, an increase in CP into adulthood could not be identified because of the age range of participants within this study. As such, we were not able to reliably classify this group as an adolescent onset, or persistent CP group. Previous studies have also shown mixed results regarding such intermediate antisocial groups.^4^

Cross-sectional studies have shown structural brain abnormalities related to CD.^8, 9, 38^ However, the relationship between development of such neurobiological deficits and developmental pathways of CPs has remained unclear. Results indicated that the OFC showed similar cortical thinning between CP groups, suggesting that development of the OFC does not have a specific role in the developmental pathway of CP. Development of other prefrontal regions, however, did distinguish between groups. The stable low CP group, showing normative CP during adolescence, would be expected to show typical normative brain development. Indeed, this group showed a normative pattern of cortical thinning similar to patterns observed during typical adolescence.^39^ When comparing groups on their pattern of cortical development, there was less thinning of the dl-PFC and ACC over time in the desisting CP group specifically. This may indicate that the decline in gray matter^39, 40, 41^ has set in at a latter time compared with the intermediate and stable low CP groups, or an attenuation of such decline. Results from the insula suggested the lack of cortical thinning for the desisting group as seen in the other CP groups. However, this result did not survive correction for multiple comparisons.

Furthermore, the CP groups did not show differential development of subcortical volume in the amygdala. Research has shown the amygdala is involved in CD;^8^ however, current results indicated that development of this structure does not have a specific role in the developmental pathway of CP. For the hippocampus, results indicated that the volume of the right hippocampus shows differential trajectories for the CP group, in which the desisting CP group showed a deviant trajectory. This indicates that subcortical volume of the hippocampus might have a role in CP trajectories during adolescence. This structure has also been implicated in depression within the current cohort,^31^ where hippocampal growth was greater in the depression group. The comorbidity of internalizing problems and CPs has been shown in previous literature,^42^ although it has been suggested that *direct* comorbidity is uncommon.^43^ To investigate whether our current results were specific to CPs, we included analysis correcting for longitudinal internalizing problems (that is, depression and anxiety symptoms). These results showed that the differences found in the CP trajectories for cortical thickness and subcortical volume remained after correcting for internalizing problems, with the exception that some effects were reduced to trends. This may very well be caused by a reduction in power as a result of adding covariates in our GEE models. As such, our results seem specific to the trajectories of CPs and cannot be explained by the presence of internalizing problems. However, research suggests that children with antisocial behaviors develop anxiety problems later in life,^44^ and comorbidity between internalizing problems and antisocial behaviors may involve common risk factors.^45^ As such, future studies would benefit from investigating the co-occurrence and a more complicated interplay between the development of internalizing and antisocial behaviors.

In addition, analyses indicated that the desisting CP group showed distinct trajectories of surface area in the insula and gyrification in the ACC (see Supplementary Data). Differences between CP groups regarding the trajectories of surface area and gyrification seemed to involve less widespread differences compared with cortical thickness. Overall, current results are in line with earlier (cross-sectional) imaging studies, showing that frontal and temporal areas are related to CPs.^5, 6, 16^ It has been suggested that CD might reflect a delay in cortical maturation.^9^ However, neurodevelopment can go awry in several ways^19^ and we were not able to distinguish between pathological trajectories. Studies have shown that attenuations in cortical brain development are related to adolescent psychopathology.^19, 31^ Interestingly, deviant cortical maturation seemed to specifically underlie a desisting CP trajectory, as opposed to an intermediate CP trajectory. Possibly, a desisting behavioral outcome is related to deviant, but eventually normalizing, cortical thickness in antisocial adolescents as has been shown in ADHD.^19^

Research has suggested that interactions between prefrontal and cingulate control systems are involved in regulating emotion.^46^ The insular cortex is linked to autonomic regulation, and is thought to support integration of body and mind.^47^ Furthermore, cognitive control is thought to be a dynamic process involving interactions between the dl-PFC and ACC, in which the dl-PFC provides input regarding more strategic aspects of cognitive control, whereas the ACC likely involves evaluative processes indicating when such control needs to be more (or less) engaged.^47^ Indeed, there is increasing agreement on deficits in emotion recognition and emotional reactivity being related to CPs in adolescents.^48^ Current results suggest that neurodevelopmental disturbances in the dl-PFC, ACC and insula may cause such deficits in emotion, emotion control and autonomic regulation leading to CPs in early adolescence. As such, a desisting CP trajectory is possibly related to delayed but eventually normalizing neurodevelopmental disturbances (as observed in ADHD^19^) in areas involved in emotion and emotional control.

Recent research has shown that genes have a substantial role in antisocial behavior.^49^ Interestingly, it has been shown that the development of cortical thickness throughout the lifespan is closely related to genetic influences.^40^ As such, the distinct trajectories of cortical thickness related to CP development might (partly) involve shared genetics. Such a relationship likely involves (complicated) gene–environment interactions, meaning that there is an interplay between the environment, genes and behavioral outcome. Recently, Weeland *et al.*^50^ proposed that in gene by environment interactions (G × E), in relation to development of antisocial behavior, the genetic make-up would either strengthen or weaken the effect of environment (for example, family adversity). Furthermore, this moderation is in turn explained or moderated by a biophysical trait such as emotional reactivity, rewards or punishment sensitivity. As such, antisocial behavior possibly involves a complex interplay where genes influence cortical development, which may moderate emotional reactivity and control, in turn moderating the behavioral outcome (for example, CP trajectories). Current results indicated that a desisting CP trajectory involved deviant cortical development. As such, this may involve G × E interactions promoting resilient development^51^ or indicate a positive emotional environment. Unfortunately, this was beyond the scope of the current study, and future studies are needed to further investigate the underlying mechanism of desisting (as well as persistent) CP trajectories.

Limitations should be noted, as mentioned earlier, that this study involved a possible interscanner bias. Steps were employed to reduce such bias, and former studies using the current sample indicate that interscanner bias is minimal.^31, 32^ It was shown that most individuals experienced change between assessment waves 1 and 2 greater than what would be expected from any scanner-related differences. Furthermore, as all adolescents were assessed in the same scanner at each wave, this would not influence longitudinal between-group comparisons. However, future studies would benefit from conducting all MRI assessment waves on the same scanner to remove this potential confound. Furthermore, the desisting CP group consisted of a small group (*n*=13). This issue is related to the nature of such antisocial behaviors, as severe early-onset antisocial groups are not prevalent.^3, 4^ However, this has an impact on the power of the current study to detect group difference. Owing to limited numbers within the desisting CP group and co-variance conditions (that is, gender), the current study was unable to perform additional whole-brain analysis. Furthermore, we did not detect a severe persistent CP group. This might be explained by recruitment of community-based children, rather than a sample selected to maximize these traits. Persistent CP children can be expected to be involved in some form of health-care service at an early age (for example, residential treatment). As such, these children may not have been present, and it is likely that such families would not agree to participate because of problematic home situations. Future studies should aim for a higher number of participants with a desisting CP trajectory and include adolescents with a severe persistent CP trajectory. In addition, future studies should consider other analyses techniques to investigate brain development, and behavioral changes related to CPs, to fully understand the relation.

In conclusion, we believe this is the first longitudinal imaging study investigating neurodevelopmental trajectories in relation to CP pathways. Our findings indicated that a deviant cortical thickness trajectory across adolescence was related to a desisting CP pathway. This may act as an underlying mechanism for developmental CP trajectories, and possibly distinguish desisting from persisting antisocial adolescents. Future research should include a severe persistent CP group investigating the cortical development compared with desisting CP, and more MRI assessments throughout early and late adolescence. Furthermore, research on brain connectivity may further uncover neurodevelopmental abnormalities in relation to CP development.^52, 53, 54, 55^

## Figures and Tables

**Table 1:** The fit indices of the LCGA

*(A)*	*LMR-LRT*	*BLRT*	*BIC*	*Entropy*
Two classes	0.0161	<0.001	2305.228	0.858
Three classes	0.3902	<0.001	2276.989	0.850
Four classes	0.0139	<0.001	2270.388	0.889
				
*(B)*				
Two classes	0.1691	<0.001	1710.145	0.864
Three classes	0.1293	<0.001	1693.911	0.913
Four classes	0.7775	0.0128	1695.469	0.873

Abbreviations: BIC, Bayesian Information Criterion; BLRT, bootstrap likelihood ratio test; LCGA, latent class growth analysis; LMRT, Lo, Mendell and Rubin likelihood ratio test.

The table includes all data available (A) and complete data only (B).

**Table 2:** Descriptives of the three CP trajectory classes

*Class*	n	*Gender,% male*	*IQ, mean (s.d.)*	*SES, mean (s.d.)*	*Wave*	*Age, mean (s.d.)*	*sMRI,* n
Intermediate CP	37	59.5	101.0^a^ (8.6)	54.4 (19.2)	T1	12.8 (0.4)	33
					T2	16.7 (0.5)	29
					T3	19.2 (0.5)	22
							
Desisting CP	13	84.6	102.3 (11.3)	55.2 (20.7)	T1	12.8 (0.6)	12
					T2	16.8 (0.4)	8
					T3	19.2 (0.2)	4
							
Stable low CP	121	46.3	106.3^a^ (12.5)	58.3 (21.8)	T1	12.8 (0.4)	100
					T2	16.7 (0.5)	100
					T3	19.0 (0.5)	78

Abbreviations: CP, conduct problems; IQ, intelligence based on the WISC short form; SES, socioeconomic status based on the ANU4; sMRI, structural magnetic resonance imaging; WISC, Wechler Intelligence Scale for Children.

a Significant difference between the intermediate and stable low CP class (*P*<0.05).
