# Supplementary material for: Trajectories of adolescent conduct problems in relation to cortical thickness development: a longitudinal MRI study
Source: Transl Psychiatry. 2016 Jun 21;6(6):e841–. doi: 10.1038/tp.2016.111 (PMC4931609; doi:10.1038/tp.2016.111)
Supplement: Supplementary Information [file tp2016111x1.pdf]

Supplementary Data

Disruptive Behavior Disorder

Each LCGA class was checked for participants with criteria for Disruptive Behavior Disorder (Conduct Disorder and/or Oppositional Defiant Disorder) using the Schedule for Affective Disorder and Schizophrenia for School-Aged Children: Present and Lifetime Version[1] (K-SADS). This revealed that at the first assessment wave a total of 7 adolescents met DBD criteria, of which 5 in the intermediate CP group, and 2 in the desisting CP group. At the second assessment wave 3 more adolescents met DBD criteria, of which 2 from the intermediate CP group and 1 from the stable low CP group. At the third assessment wave, only 2 adolescents still met the DBD criteria from the intermediate CP class.

Post-hoc analysis

In addition to cortical thickness of the five regions of interest presented in the manuscript, surface area (SA) and local gyrification (LGI) was investigated. The regression model contained age at measurement and LCGA class as independent variables, together with their interaction. The main interest was in testing whether changes over time differed between the classes by testing the interaction for significance. Separate GEE analyses were performed for different ROIs, left and right separately. Significance level was set at 5% for all analyses. All analyses were corrected for SES, gender and the interaction between gender and age to account for gender and SES-related differences in mean cortical thickness and gender-related differences in changes in thickness over time. Please note that for the LGI analyses, 11 cases were discarded (five for the left hemisphere

and six for the right hemisphere) as the pipeline failed to generate local gyrification index map due to topological defects.

Results

Surface area

Changes in surface area in the left and right OFC, dl PFC and the ACC over time did not differ between the CP groups. In addition, changes in the right insula and the left and right parahippocampal area showed no differences over time between CP groups. For the left parahippocampal area there was a significant interaction effect gender and age (Wald's, $\chi 2 = 5.36$, df=1, $p<.05$). A significant interaction between age and CP group was found for the left insula (Wald's, $\chi 2 = 10.97$, df=2, $p<.005$). Plotted results for the left insula for the three CP groups indicated a greater decrease for the desisting CP group compared to the other groups (See figure 2). After applying a Bonferonni correction for multiple comparisons ($p<.005$), the interaction effect of age and class for the insula remained significant.

Local Gyrification

Changes in local gyrification in the left and right OFC, dl PFC and the left ACC over time did not differ between the CP groups. In addition, changes in the left and right insula and parahippocampal area showed no differences over time between CP groups. For the left insula (Wald's, $\chi 2 = 13.90$, df=1, $p<.000$) and the right dl PFC (Wald's, $\chi 2 = 5.82$, df=1, $p<.05$) there was a significant interaction effect gender and age. A significant interaction between age and CP group was found for the right ACC (Wald's, $\chi 2 = 10.95$, df=2, $p<.005$). The desisting CP group

showed reduced increase in local gyrification over time in comparison to the other CP groups (see Figure S1). After applying a Bonferonni correction for multiple comparisons ($p<.005$), the interaction effect of age and class for the ACC remained significant.

[Figure S1 supplementary data]

Discussion

The present supplementary post-hoc analyses were mostly in line with our main results of the paper involving cortical thickness trajectories. The CP groups did show differential trajectories of surface area in the insula and differential trajectories for gyrification in the ACC. Similar to results for cortical thickness, deviant cortical growth trajectories were observed in the desisting CP group as compared to the other two groups (see Figure S2). However, differences between CP groups regarding the trajectories of surface area and gyrification seemed to involve less widespread differences compared to cortical thickness. Research has indicated that surface area, gyrification and cortical thickness are (at least party) biologically distinct morphological properties[4–6]. As such, different cellular mechanisms might be implicated in the different brain areas involved. Future research would benefit from focusing on all three morphological properties and the relation to CP trajectories, to further investigate which specific mechanism are involved.

References

1 Kaufman J, Schweder AE. The Schedule for Affective Disorders and Schizophrenia for School-Age Children: Present and Lifetime version (K-SADS-PL). In: *Comprehensive handbook of psychological assessment, Vol. 2: Personality assessment.* 2004, pp 247–255.

2 Fairchild G, Passamonti L, Hurford G, Hagan CC, von dem Hagen EAH, van Goozen SHM *et al.* Brain structure abnormalities in early-onset and adolescent-onset conduct disorder. *Am J Psychiatry* 2011; **168**: 624–33.

3 Whittle S, Lichter R, Dennison M, Vijayakumar N, Schwartz O, Byrne ML *et al.* Structural Brain Development and Depression Onset During Adolescence: A Prospective Longitudinal Study. *Am J Psychiatry* 2014; **171**: 564–571.

4 Wierenga LM, Langen M, Oranje B, Durston S. Unique developmental trajectories of cortical thickness and surface area. *Neuroimage* 2014; **87**: 120–126.

5 Hogstrom LJ, Westlye LT, Walhovd KB, Fjell AM. The structure of the cerebral cortex across adult life: age-related patterns of surface area, thickness, and gyrification. *Cereb Cortex* 2013; **23**: 2521–30.

6 Sanabria-Diaz G, Melie-García L, Iturria-Medina Y, Alemán-Gómez Y, Hernández-González G, Valdés-Urrutia L *et al.* Surface area and cortical thickness descriptors reveal different attributes of the structural human brain networks. *Neuroimage* 2010; **50**: 1497–510.
