# Supplementary figures and images for: Trajectories of adolescent conduct problems in relation to cortical thickness development: a longitudinal MRI study
Source: Transl Psychiatry. 2016 Jun 21;6(6):e841–. doi: 10.1038/tp.2016.111 (PMC4931609; doi:10.1038/tp.2016.111)

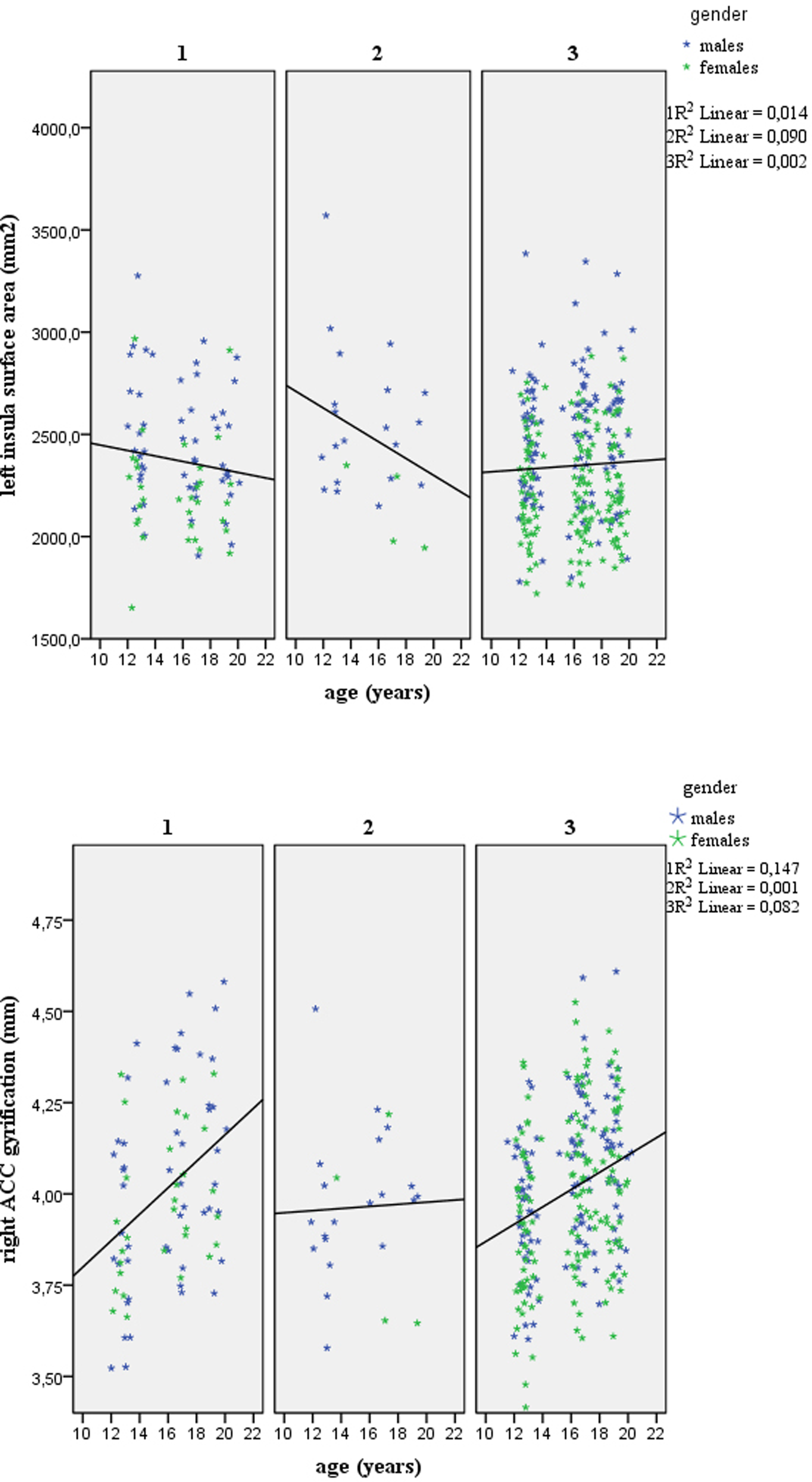

Supplement: Supplementary Figure 1 [file tp2016111x2.tif]
